# The food effect on the pharmacokinetics of TQB3616 capsule in Chinse healthy subjects: a randomized, open-label, single-center, two-period, two-sequence crossover phase I clinical trial

**DOI:** 10.3389/fphar.2025.1586368

**Published:** 2025-05-30

**Authors:** Jun Dai, Yanan Zhou, Lei Zhang, Qing Ren, Zhengzhi Liu, Yanli Wang, Yang Cheng, Qiaohuan Deng, Haimiao Yang, Hong Zhang

**Affiliations:** ^1^ School of Life Science and Biopharmaceutics, Shenyang Pharmaceutical University, Shenyang, China; ^2^ The Affiliated Hospital of Changchun University of Traditional Chinese Medicine, Changchun, China

**Keywords:** TQB3616, breast cancer, human epidermal growth factor receptor, hormone receptor, food-effect studies

## Abstract

**Purpose:**

The purpose of this study was to evaluate the food effect on the pharmacokinetics of TQB3616 capsule in Chinse healthy subjects.

**Methods:**

The subjects were randomly allocated to two distinct sequences in a 1:1 ratio. During each treatment periods, subjects ingested a single oral dose of 180 mg TQB3616 capsule administered with 240 mL of warm water under fasted and fed conditions. To avoid carryover effects, a 19 days washout period was strictly implemented between treatment periods. Blood samples were collected in accordance with the study protocol, and the plasma concentration of TQB3616 was measured using a fully validated liquid chromatography-tandem mass spectrometry (LC-MS/MS) method. Safety evaluations were performed continuously throughout the duration of the trial.

**Results:**

Following the administration of TQB3616 capsules under both fasted and fed conditions, the geometric mean ratios of C_max_, AUC_0-t_, and AUC0- ∞ for TQB3616 in the fed state compared to the fasted state were 148.04%, 145.06%, and 143.13%, respectively. The corresponding 90% confidence intervals (CIs) were 101.23%–216.51%, 117.68%–178.83%, and 116.46%–175.91%, none of which fell within the conventional bioequivalence range of 80.00%–125.00%. A total of 81 adverse events (AEs) were reported among 16 subjects, with 77 events deemed related to the drug. Among the 77 drug-related adverse events, two cases were grade II, with the rest being grade I. Notably, there were no serious adverse events, deaths, or unexpected serious reactions.

**Conclusion:**

A pharmacokinetic study conducted on healthy volunteers, who received a single 180 mg dose of TQB3616 capsules under fasting and fed conditions, demonstrated clinically significant effects of food on the drug’s bioavailability. Compared with fasted, postprandial administration delayed median T_max_ by 1 h while increasing total systemic exposure and peak concentration. Additionally, postprandial dosing was associated with reduced incidence of gastrointestinal adverse reactions. These data support the recommendation that TQB3616 capsules be administered with food to maximize therapeutic bioavailability while improving gastrointestinal tolerability profile.

**Clinical Trial Registration:**

http://www.chinadrugtrials.org.cn, identifier, CTR20210354; clinicaltrials.gov, identifier, NCT05344729.

## 1 Introduction

Breast cancer is the most common malignancy and the leading cause of cancer-related mortality among women globally. In 2022, an estimated 2.3 million new cases and 665,684 deaths were reported, representing 11.6% of all female cancer diagnoses and 6.9% of cancer-related fatalities, thereby establishing it as the foremost contributor to both incidence and mortality in this population (Bray et al., 2024). Metastasis remains the primary driver of breast cancer-associated deaths (Tallón de Lara et al., 2021; Wu et al., 2023), with 3%–10% of newly diagnosed patients exhibiting distant metastatic disease at initial presentation (Zhong et al., 2023). Furthermore, 30%–40% of early-stage cases eventually progress to advanced breast cancer, which carries a 5-year survival rate of merely 20% (Zhong et al., 2023; Gonzalez-Angulo et al., 2007). Clinically, breast cancer is classified into three major subtypes: hormone receptor-positive (HR+), human epidermal growth factor receptor two-positive (HER2+), and triple-negative (TN) breast cancer (Goldhirsch et al., 2011; Natori et al., 2023). HR + breast cancer, accounting for approximately 70% of cases, represents the most prevalent subtype (Razavi et al., 2020; Nair et al., 2022). While endocrine therapy remains the cornerstone of treatment for HR + metastatic breast cancer, the development of resistance significantly compromises its long-term efficacy, posing a critical therapeutic challenge (Pernas et al., 2024).

Palbociclib, a first-in-class cyclin-dependent kinase 4/6 (CDK4/6) inhibitor developed by Pfizer, marked a paradigm shift in HR + breast cancer management. In 2015, the U.S. Food and Drug Administration (FDA) granted approval for palbociclib in combination with letrozole, a third-generation aromatase inhibitor, as first-line therapy for postmenopausal women with HR+/HER2-advanced breast cancer (Pernas et al., 2024; Finn et al., 2015). Subsequently, the FDA expanded its approval to include palbociclib combined with fulvestrant for patients who developed resistance to prior endocrine therapies (IBRANCE palbociclib [package insert], 2017). These advancements have significantly enhanced clinical outcomes, offering prolonged progression-free survival and improved quality of life for individuals with HR + metastatic breast cancer (Foffano et al., 2024).

TQB3616 capsule is a small-molecule oral drug developed by Chia Tai Tianqing Pharmaceutical Group Co., Ltd. for inhibiting cyclin-dependent kinases four and 6 (CDK4/6). Its primary mechanism of action is to reduce the phosphorylation levels of retinoblastoma protein (Rb) in cancer cells, thereby blocking the cell cycle progression from the Gap 1 (G1) phase to the Synthesis (S) phase, and thus inhibiting the proliferation of tumor cells. It is clinically intended for the treatment of recurrent/metastatic breast cancer that is HR+ and HER2-.

Preclinical studies (unpublished data) have demonstrated that TQB3616 exerts potent inhibitory effects on CDK4/6 kinase activity. Furthermore, these studies showed that TQB3616 selectively inhibits proliferation of HR+/HER2-breast cancer MCF-7 cells *in vitro* and suppresses MCF-7 tumor growth *in vivo*. In a human MCF-7 xenograft model, TQB3616 achieved comparable anti-tumor efficacy to Palbociclib at reduced dosage levels. An early phase I clinical trial (randomized, open-label, single-center, two-period and two-sequence crossover design) was conducted to evaluate the food effect on TQB3616 pharmacokinetics in healthy subjects.

## 2 Materials and methods

### 2.1 Materials and study population

The test formulation, TQB3616 (Specification: 5 mg/capsule, Batch NO.: 191,226,128, Expiry Date: 2021.12.25), was manufactured and supplied by Chia Tai Tianqing Pharmaceutical Group Co., Ltd.

This trial enrolled healthy male and female subjects aged 18–65 years, with a body mass index (BMI) ranging from 18 to 28 kg/m^2^, and a weight of ≥50 kg (males) and ≥45 kg (females). The subjects enrolled in the study were in clinical healthy, exhibiting no prior history of psychiatric abnormalities or diseases involving the cardiovascular, nervous, respiratory, digestive, urinary, or endocrine systems, no prior history of metabolic disorders. Subjects were excluded from the study if they met any of the following criteria: a history of arterial or venous thrombotic events within the preceding 6 months, including cerebrovascular accidents (such as transient ischemic attacks), deep vein thrombosis, or pulmonary embolism; conditions that could impair oral medication intake, such as dysphagia or gastrointestinal disorders; use of any prescription or over-the-counter medications, vitamin supplements, or herbal products within 1 month prior to the administration of the study drug; use of CYP3A4 inhibitors or inducers within 1 month prior to screening or the study drug administration; consumption to special diets (including grapefruit consumption) or engagement in strenuous physical activity within 14 days prior to screening; or any other factors potentially influencing the absorption, distribution, metabolism, or excretion of drugs; presence of clinically significant laboratory abnormalities during the screening period; donation of blood or experience of significant blood loss (exceeding 450 mL) within 3 months prior to administration of the study drug; participation in any clinical drug trials within 3 months prior to administration of the study drug; smoking more than five cigarettes per day within 3 months prior to the study; a history of excessive alcohol consumption within 2 weeks prior to screening, defined as consuming 14 units of alcohol per week or positive results on an alcohol breath test (1 unit = 360 mL of beer, 45 mL of 40% alcohol spirits, or 150 mL of wine); positive drug screening results or a history of drug use within 3 months prior to the study; inability to tolerate venous blood draws or presence of poor vascular conditions; inability to complete the study due to personal reasons; and any other reasons deemed by the investigators to render the subject unsuitable for enrollment. After a full understanding of the trial procedures and potential side effects, participants provided their informed consent. They also confirmed no pregnancy plans from 2 weeks prior to the administration of the investigational drug until at least 6 months following the final dose, and voluntarily committed to using effective contraceptive measures.

### 2.2 Study process

This was a randomized, open-label, single-center, two-period, two-sequence crossover early phase I clinical trial to evaluate the food effect on the pharmacokinetics of TQB3616 capsules in healthy subjects. The trial was conducted at Affiliated Hospital of Changchun University of Chinese Medicine and the protocol was approved by the Ethics Committee of the Affiliated Hospital of Changchun University of Chinese Medicine (CCZYFYLL 2021-006). The ethical approval process of this study complied with the requirements of Good Clinical Practice (GCP), the Declaration of Helsinki and the relevant domestic laws and regulations. All subjects had provided written informed consent before the study.

Subjects were admitted to the research center 2 days prior to drug administration and randomly assigned to either Group A or Group B at a 1:1 ratio. Group A, subjects received the study drug under fasted condition in period one and under fed condition in period 2. Conversely, in Group B, subjects received the study drug under fed conditions in the first period and under fasting conditions in the second period. The study employed a crossover design, with a washout period of at least 19 days between the two treatment periods.

The study protocol mandated that subjects take the medication after an overnight fast lasting at least 10 h. In a fasted condition, subjects consumed a 180 mg TQB3616 capsule with 240 mL of warm water, ensuring they remained and fasted for an additional 4 h post-dose. In a fed condition, subjects consumed a 180 mg TQB3616 capsule with 240 mL of warm water after consuming a high-fat, high-calorie breakfast (approximately 800–1,000 kcal, Supplementary table) 30 min prior to adminstration, subjects began their breakfast and completed the meal within the next 30 min. Capsules were swallowed whole without chewing.

### 2.3 Biological samples collection and process

Pharmacokinetic (PK) samples were collected at specific times: 0 h (within 60 min prior to dosing), 1 h, 2 h, 4 h, 5 h, 5.5 h, 6 h, 6.5 h, 7 h, 7.5 h, 8 h, 12 h, 24 h, 48 h, 72 h, 120 h, 168 h and 264 h post-dosing, totaling 18 sampling points. At each scheduled time, approximately 3 mL of blood was collected with a pre-chilled EDTA-K2 tubes. After gentle 180° inversion (×4) for homogenization, samples were immediately transferred to an ice bath. All blood specimens were centrifuged at 3,500 rpm for 10 min within 30 min of collection. The obtained plasma was aliquoted and stored at −80°C ± 10°C until batch analysis.

### 2.4 Drug concentration determination and method validation

Plasma concentrations of TQB3616 were quantified using a validated liquid chromatography-tandem mass spectrometry (LC-MS/MS) method. Calibration standards and quality control samples (K_2_-EDTA plasma matrix) were prepared using [D_8_]-TQB3616 as the internal standard. Protein precipitation extraction from 100 μL plasma was performed followed by chromatographic separation on a C18 column (50 × 2.1 mm, 1.7 μm). Mass transitions m/z 447.1→431.3 (TQB3616) and m/z 455.2→439.3 (IS) were monitored for quantification.

This study undertook a comprehensive validation of the LC-MS/MS methodology for quantifying TQB3616. The validated quantitative range for TQB3616 was established between 0.3 ng/mL and 300 ng/mL. The validation results indicated that the method is characterized by high sensitivity, selectivity, accuracy, and reproducibility. Moreover, TQB3616 demonstrated satisfactory stability under various conditions: it remained stable at room temperature for at least 23.5 h, endured four freeze-thaw cycles, and was stable for 89 days at both −20°C and −80°C. Sample dilution integrity was confirmed at 20-fold dilution.

### 2.5 Safety analysis

The safety analysis utilized the safety data set (SS), comprising all subjects receiving TQB3616 with recorded safety assessments. The safety assessments indicators included adverse events, serious adverse events, laboratory tests (routine blood, blood biochemistry, urinalysis, and coagulation), vital signs (complete blood count, serum biochemistry, routine lipid tests, urinalysis), 12-lead electrocardiogram, and physical examination. The frequency, type, extent, and comorbid medications of all treatment-emergent adverse events (TEAEs) occurring during treatment were recorded systematically documented. For severity, adverse events were rated and documented using the National Cancer Institute-Common Terminology Criteria for Adverse Events (CTCAE) version 5.0. The Medical Dictionary for Regulatory Activities (MedDRA) was used to encode the classification of adverse events. Any adverse events observed during the trial were monitored until resolution, return to baseline levels, or stabilization the subject’s condition.

### 2.6 Statistical analysis

Phoenix WinNonlin (version 8.3.4) was used to calculate pharmacokinetic parameters, including: maximum plasma concentration (T_max_), area under the curve (AUC) from time zero to the last measurable concentration (AUC_0-t_), AUC from time zero to observed infinity (AUC0- ∞ ), peak concentration (C_max_),terminal half-life of analyte in plasma (t_1/2_), terminal rate constant (λz), and extrapolated AUC percentage (AUC_extrp_ (%)). Other statistical analyses were performed using SAS (version 9.4). All tests were considered statistically significant at *p* < 0.05.

Based on the Pharmacokinetics Parameter Set (PKPS), main parameters were summarized by fasting/fed groups using sample size, arithmetic mean, standard deviation, coefficient of variation, median, minimum, maximum, as well as geometric mean and geometric coefficient of variation.

For the food-effect equivalence analysis set (BES), a mixed-effects model analyzed natural log-transformed C_max_, AUC_0-t_, and AUC0- ∞ . With fasted as the reference, the geometric mean ratios and 90% confidence intervals (CIs) were calculated. The pharmacokinetics equivalence was concluded if the 90% CIs for C_max_, AUC_0-t_, and AUC0- ∞ fell within 80.00%–125.00%.

## 3 Results

### 3.1 Baseline demographic and characteristics

**FIGURE 1:**
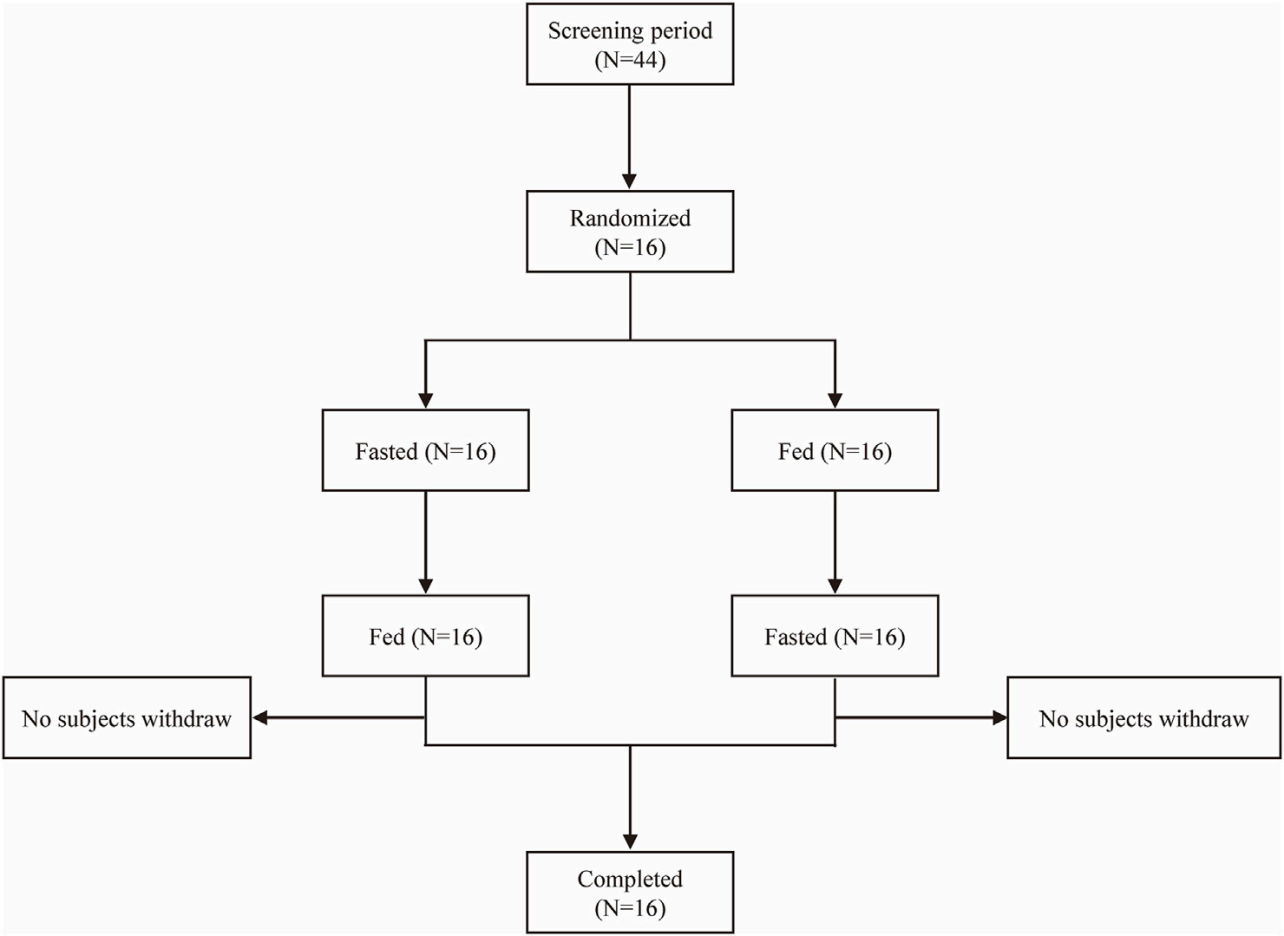
Study flow diagram.

A total of 44 subjects were screened, and 16 eligible subjects participated in the study and completed both treatment periods according to the protocol (Figure 1). Subjects with random numbers r002, r006, r009, r010, and r012 were excluded from the PKPS and BES in the fasting period due to vomiting between 0 and 2 times the median T_max_ after dosing. Subjects with random numbers r001, r006, and r009 were excluded from the PKPS and BES in the fed period. The age of all subjects ranged from 35 to 57 years; body weight from 47.0 kg to 80.6 kg; and body mass index (BMI) from 19.1 kg/m^2^ to 26.8 kg/m^2^. The population and baseline characteristics of the subjects met the inclusion criteria and did not meet the exclusion criteria. Table 1 showed the demographics and characteristics of the participants.

**TABLE 1:** The demographics and features of the participants in the study.

Statistic		Group A (N = 8)	Group B (N = 8)	Total (N = 16)
Age (year)	N	8 (0)	8 (0)	16 (0)
	Mean (SD)	48.6 ± 4.57	44.8 ± 7.11	46.7 ± 6.11
	Median (Q1, Q3)	49.0 (46.0, 53.0)	45.0 (39.5, 48.5)	47.0 (44.0, 51.0)
	Min-Max	40-53	35-57	35-57
Gender	Male	5 (62.5)	5 (62.5)	10 (62.5)
	Female	3 (37.5)	3 (37.5)	6 (37.5)
Hight (cm)	N	8 (0)	8 (0)	16 (0)
	Mean (SD)	160.81 ± 7.416	165.81 ± 7.755	163.31 ± 7.771
	Median (Q1, Q3)	163.00 (159.25, 163.50)	166.25 (159.75, 171.50)	163.00 (159.75, 168.75)
	Min-Max	145-170	154.5-177	145-177
Weight (kg)	N	8 (0)	8 (0)	16 (0)
	Mean (SD)	64.36 ± 6.956	64.29 ± 10.103	64.33 ± 8.379
	Median (Q1, Q3)	63.45 (59.85, 69.50)	63.80 (59.25, 70.30)	63.80 (59.85, 69.65)
	Min-Max	54.1-75.2	47-80.6	47-80.6
BMI (kg/m^2^)	N	8	8	16
	Mean (SD)	24.8 (1.4)	23.3 (2.1)	24.1 (1.9)
	Median (Q1, Q3)	25.1 (23.9, 25.9)	23.2 (22.9, 24.1)	24.1 (22.9, 25.5)
	Min-Max	22.5-26.7	19.1-26.8	19.1-26.8

Data are presented as mean ± SD, or n (%).

BMI: body mass index: weight/(height in meters)^2^, N number, SD, standard deviation.

### 3.2 Pharmacokinetic results

**FIGURE 2:**
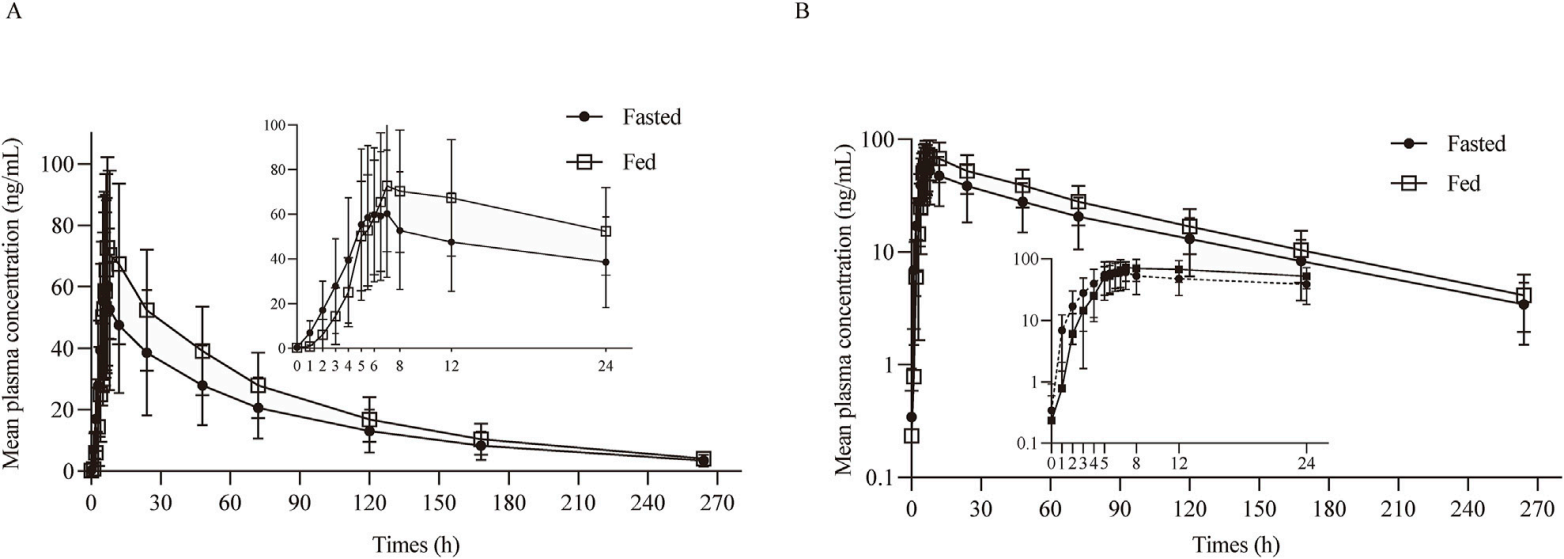
PK analysis of TQB3616. Mean blood concentration (±SD) time curve after oral administrated TQB3616 under fasted and fed condition **(A)** and log transformation **(B)**.

After oral administration a TQB3616 capsule, the mean ± SD plasma drug concentration-time curve was shown in Figure 2A. The curve after logarithmic transformation was shown in Figure 2B. The time of the maximum concentration under fasting and fed conditions were 6.0 h and 7.0 h, respectively. The C_max_ values of TQB3616 under fasting and fed conditions were 60.39 ± 37.17 ng/mL and 75.39 ± 28.34 ng/mL, respectively. The AUC_0-t_ values were 3,634.80 ± 2,174.18 h·ng/mL and 5305.30 ± 1948.11 h·ng/mL, respectively. The AUC0- ∞ values were 3,920.86 ± 2,325.20 h·ng/mL and 5710.59 ± 2,100.68 h·ng/mL, respectively. Other pharmacokinetic parameters were shown in Table 2.

**TABLE 2:** The PK parameters of TQB3616.

PK parameters (unit)	Mean ± SD (N = 16)	
	Fasted (n = 11)	Fed (n = 13)
C_max_ (ng/mL)	60.39 ± 37.17	75.39 ± 28.34
^a^T_max_(h)	6.0 (5.0, 24.0)	7.0 (7.0, 12.0)
AUC_0-t_ (h^a^ng/mL)	3,634.80 ± 2,174.18	5305.30 ± 1948.11
AUC0- ∞ (h^a^ng/mL)	3,920.86 ± 2,325.20	5710.59 ± 2,100.68
AUC__Extrap_ (%)	7.57 ± 3.29	6.97 ± 2.90
t_1/2_(h)	71.27 ± 11.83	68.95 ± 10.68
λ_Z_ (h^-1^)	0.01 ± 0.002	0.01 ± 0.002

^a^ T_max_: represented as the median (min, max).

N: the number of subjects in the pharmacokinetic parameter analysis set.

n: the number for statistical analysis.

With fasted as the reference, the GMR of C_max_, AUC_0-t_, and AUC0- ∞ for TQB3616 under fasting and fed conditions were 148.04%, 145.06%, and 143.13%, respectively (Table. 3). The 90% confidence intervals (CIs) were 101.23%–216.51%, 117.68%–178.83%, and 116.46%–175.91%, respectively. The GMR of the primary endpoint PK parameters C_max_, AUC_0-t_, and AUC0- ∞ did not fall within the range of 80.00%–125.00%. Administration of the drug under fed condition had an impact on the pharmacokinetic parameters compared to fasted condition.

**TABLE 3:** Results of the equivalence determination of TQB3616 in fasted and fed condition.

PK parameters (unit)	GeoMean						
	n	Fed	n	Fasted	Fed/Fasted Ratio (%)	Ratio 90% CI (%)	CV (%)
C_max_ (ng/mL)	13	71.76	11	48.47	148.04	101.23-216.51	53.42
AUC_0-t_ (h*ng/mL)	13	4792.51	11	3,303.72	145.06	117.68-178.83	26.51
AUC0- ∞ (h*ng/mL)	13	5130.98	11	3,584.79	143.13	116.46-175.91	26.07

n: the number for statistical analysis.

CV: coefficient of variation.

### 3.3 Safety results

Among the 16 subjects who entered the safety analysis set, a total of 81 adverse events (AEs) occurred in all 16 subjects, of which 77 were considered related to the drug. Among the 77 AEs considered drug-related, except for one case of Grade II elevated total bilirubin in the fed period of subject 01,006 and one case in the fasted period of subject 01,029, all others were Grade I. Except for the AE of thrombocytopenia in subject 01,030, which was reported retrospectively and not followed up to resolution, and the AE of elevated triglycerides in subject 01,034, which was not followed up to resolution due to loss to follow-up, all AEs resolved to normal or improved without intervention. No serious adverse events, deaths, or unexpected serious adverse reactions occurred in this clinical study (Table 4).

**TABLE 4:** Summary of adverse events during the treatment period.

	Fasted		Fed	
	n	N	n	N
Total AEs	43	15	38	14
AEs related to the study drug	40	14	37	14
Serious Adverse Event	0	0	0	0
SAE related to the study drug	0	0	0	0
AEs of grade 1	39	14	36	14
AEs of grade 2	1	1	1	1
AEs of grade 3 and above	0	0	0	0

AEs: adverse event.

n: the instance of adverse event; N: the number of subjects.

## 4 Discussion

This trial, as a phase I study, focuses on evaluating the effect of food on the pharmacokinetic parametersTQB3616. In the Phase I monotherapy tolerability and pharmacokinetics clinical trial of TQB3616 (unpublished data), pharmacokinetic assessments were conducted for both single and multiple dosing across various dose groups. The T_max_ for TQB3616 was approximately 6 h–8 h, while the t_1/2_ ranged from 65 h to 70 h. Within the dose range of 20–150 mg, human exposure increased with the dose escalation after a single administration, with the exposure increase ratio slightly exceeding the dose increase ratio. Steady-state plasma concentrations were achived after approximately 7 days of continuous dosing. After 28 days of administration in the first cycle, the mean C_max_ and AUC were 3–5 times higher than those observed after a single dose, indicating significant accumulation. Based on preclinical data (unpublished data), the recommended dose of TQB3616 is 180 mg twice daily. Consistent with regulatory requirements for food-effect studies, the highest intended clinical dose (180 mg) was selected for this bioavailability assessment. The washout period of 19days was established, a duration seven times longer than the drug’s metabolic half-life, ensuring that the drug concentration subsequent administration fell below the lower limit of quantitation for the bioassay. In accordance with the FDA’s 2002 Guidance for Industry on Food-Effect Bioavailability and Fed Bioequivalence Studies, which recommends a minimum of 12 subjects to complete the study, we enrolled 16 subjects to account for potential dropouts (Assessing the Effects of Food).

The study results indicated that, compared with the fasted state, food intake delayed the absorption rate of TQB3616 while significantly enhancing its extent of absorption. Specifically, food intake increased the C_max_ of TQB3616 by 24.84%, and the AUC_0-t_ and AUC0- ∞ by 45.95% and 45.65%, respectively. The difference in C_max_ between the fasted and fed states was not statistically significant (*p* > 0.05), whereas the differences in AUC_0-t_ and AUC0- ∞ were statistically significant (*p* < 0.05). The AUC, an indicator of total drug exposure within the body, showed significant changes, suggesting that the effect of food on the overall bioavailability of TQB3616 exceeds its effect on C_max_. Consequently, the food effect on the total amount absorbed is more substantial. Food intake delayed the absorption of TQB3616, with the median T_max_ prolonged by approximately 1 h. The difference in T_max_ between the fasted and fed states was statistically significant (*p* < 0.05). The terminal elimination half-life of TQB3616 was slightly shortened, with values of 71.27 ± 11.83 h in the fasted state and 68.95 ± 10.68 h in the fed state. These PK changes suggest a potential enhancement in efficacy due to increased drug exposure under fed conditions. However, given the limited sample size and the study being conducted in healthy volunteers, the clinical relevance of these PK differences remains uncertain. Notably, postprandial administration was associated with faster recovery from gastrointestinal (GI) AEs and a lower incidence of GI-related AEs, although the difference in AE rates was not statistically significant. These findings suggest that taking TQB3616 with food may improve tolerability by reducing GI irritation. The observed food effect on PK parameters is consistent with other CDK4/6 inhibitors (Ruiz-Garcia et al., 2014; Liu et al., 2022).

The safety profile of the drug was favorable in both fasted and fed states, with adverse reactions primarily involving gastrointestinal disorders such as diarrhea, abdominal discomfort, and emesis. Taking the medication after meal may reduce gastrointestinal side effects, aligning with the preclinical results of TQB3616. In preclinical studies of TQB3616 (unpublished data), gastrointestinal toxicity and hematological/serum biochemical abnormalities were observed at medium-to-high dose levels. These findings align with reported clinical data on CDK4/6 inhibitors (including gastrointestinal reactions, hematological toxicity, and elevated liver enzymes) (IBRANCE palbociclib [package insert], 2017; Ruiz-Garcia et al., 2014; Liu et al., 2022; Ji et al., 2020), indicating that the drug-related adverse events (AEs) documented in this study possess potential clinical relevance. Notably, all treatment-emergent AEs following single oral administration of TQB3616 resolved spontaneously without medical intervention.

During the fasted treatment period, a total of 43 adverse events were documented. Adverse events with an incidence rate exceeding 5% included diarrhea, abdominal discomfort, nausea, emesis, and elevated blood triglyceride levels, as detailed in Table 5. The most frequently reported adverse events were gastrointestinal. On average, these gastrointestinal reactions occurred 4.2 h (range: 1.7–7.3 h) post-dosing and resolved spontaneously without intervention within an average of 3.2 h (range: 0–10 h post-dosing. During the fed treatment period, a total of 38 adverse events were documented. Adverse events with an incidence rate exceeding 5% included diarrhea and abdominal discomfort, as detailed in Table 5. The most frequently reported adverse events were gastrointestinal. On average, these gastrointestinal reactions manifested 4.3 h (range: 2.1–8.4 h) post-dosing and resolved spontaneously without intervention within an average of 2.2 h (range: 0–9.8 h) post-dosing. In this clinical trial, a single-dose regimen was used, and the main adverse events observed were diarrhea, abdominal discomfort, and emesis, which were similar to the results of previous studies on this product (unreported). However, the possibility of other factors influencing these results cannot be ruled out.

**TABLE 5:** The summary of Treatment-Emergent Adverse Events by System Organ Class during the treatment period.

System Organ class	Fasted		Fed	
	n (%)	N	n	N
Gastrointestinal Disorders	32 (39.53%)	12	27 (33.33%)	13
Diarrhea	11 (13.58%)	11	10 (12.35%)	10
Abdominal discomfort	6 (7.42%)	6	9 (11.11%)	9
Nausea	6 (7.42%)	6	3 (3.7%)	3
emesis	5 (6.17%)	5	3 (3.7%)	3
Abdominal pain	4 (4.94%)	4	2 (2.47%)	2
laboratory tests	11 (13.58%)	9	10 (12.33%)	5
Blood triglycerides increased	6 (7.42%)	6	4 (4.94%)	4
Blood bilirubin increased	1 (1.23%)	1	1 (1.23%)	1
Protein total decreased	1 (1.23%)	1	1 (1.23%)	1
Urinary occult blood positive	0	0	2 (2.47%)	1
Blood bilirubin unconjugated increased	0	0	1 (1.23%)	1
Thrombocyte count decreased	1 (1.23%)	1	0	0
White blood cells urine positive	2 (2.47%)	1	0	0
Red blood cells urine positive	0	0	1 (1.23%)	1
Nervous System Disorders	0	0	1 (1.23%)	1
Head discomfort	0	0	1 (1.23%)	1

This study observed a relatively high C_max_ coefficient of variation (CV%: ∼53%), consistent with reported ranges for other CDK4/6 inhibitors in healthy volunteer food-effect studies (Ruiz-Garcia et al., 2014; Liu et al., 2022). Potential contributing factors included: the food-effect evaluation included both fasting and fed conditions, which inherently increases variability in absorption kinetics; as a CDK4/6 inhibitor, TQB3616’s pH-dependent solubility may contribute to inter-individual absorption differences; and the healthy subject population (n = 16) provides less variability control than larger clinical trials.

Our study identified significant gender differences in food effects, demonstrating greater postprandial increases in C_max_ and AUC for females compared to males (females: +33.5% C_max_, +69.9% AUC; males: +20.9% C_max_, +38.2% AUC), indicating more substantial food effects on drug exposure in female patients. While BMI effects remain indeterminate due to sample constraints (n = 16, limited BMI range). The current findings clearly demonstrate sex-based differences in TQB3616 pharmacokinetics following food intake, with females showing significantly greater postprandial exposure increases. To further investigate these observations, future research should include larger sample sizes and incorporate assessments of hormonal profiles and body composition to clarify the mechanisms involved. Moreover, the greater variability seen in male subjects may warrant consideration of stratified dosing approaches. These findings provide valuable insights for personalized treatment strategies and inform the design of future clinical investigations.

## 5 Conclusion

The results showed that, compared to the fasted state, taking the drug with a meal delayed absorption while increasing overall bioavailability, as indicated by a 45% rise in AUC. Furthermore, there appears to be a slight reduction in gastrointestinal adverse reactions. Consequently, it is recommended to take TQB3616 capsules with or after food.

## Data Availability

The original contributions presented in the study are included in the article/Supplementary Material, further inquiries can be directed to the corresponding authors.
