# Supplementary material for: The food effect on the pharmacokinetics of TQB3616 capsule in Chinse healthy subjects: a randomized, open-label, single-center, two-period, two-sequence crossover phase I clinical trial
Source: Front Pharmacol. 2025 May 30;16:1586368. doi: 10.3389/fphar.2025.1586368 (PMC12163317; doi:10.3389/fphar.2025.1586368)
Supplement: Supplementary file 1 [file Table1.docx]

**Supplementary Table. The composition of the high-fat high calorie meal.**

| **Ingredient** | **Content** | **Kilocalorie** | **Protein** | | **Fat** | | **Carbohydrate** | |
| --- | --- | --- | --- | --- | --- | --- | --- | --- |
|  |  | **(kcal)** | **Weight**  **(g)** | **Kilocalorie (kcal)** | **Weight**  **(g)** | **Kilocalorie (kcal)** | **Weight**  **(g)** | **Kilocalorie (kcal)** |
| Toast | 85g | 243.27 | 7.23 | 28.90 | 4.93 | 44.37 | 42.50 | 170.00 |
| Milk | 240ml | 148.80 | 6.96 | 27.84 | 8.64 | 77.76 | 10.80 | 43.20 |
| butter | 30g | 223.11 | 0.18 | 0.72 | 24.63 | 221.67 | 0.18 | 0.72 |
| sausage | 140g | 196.90 | 15.40 | 61.60 | 12.10 | 108.90 | 6.60 | 26.40 |
| egg | 100g | 143.60 | 13.30 | 53.20 | 8.80 | 79.20 | 2.80 | 11.20 |
